# Lithontriptic Action of the Uva Ursi

**Published:** 1846-02

**Authors:** 


*Lithontriptic action of the Uva Ursi.* By Dr. Fenolio.—An old calculous patient had fever, and experienced severe pain in the bladder. He would not consent to be sounded. Dr. F. prescribed a decoction of the *uva ursi*, prepared thus: ℞ Uva Ursi, ℥ss.; Water, ℥ix. Boil for fifteen minutes; strain, add syrup of gum, ʒ v., and take the whole in three doses. After using this tea for three days, the patient passed 13 pretty large gravels, and in five days more, 90 others. The whole formed a considerable mass. His suffering and fever disappeared.—*Jour. des Con. Médico-Chir.—Ibid.*

